# Study on Dynamic Modulus and Damping Characteristics of Modified Expanded Polystyrene Lightweight Soil under Cyclic Load

**DOI:** 10.3390/polym15081865

**Published:** 2023-04-13

**Authors:** Huaqiang Tao, Wenqian Zheng, Xuhui Zhou, Lin Zhou, Cuihong Li, Yanfei Yu, Ping Jiang

**Affiliations:** 1 School of Civil Engineering, Shaoxing University, Shaoxing 312000, China; taohqusx@126.com (H.T.); 21020859118@usx.edu.cn (W.Z.); 20020852108@usx.edu.cn (X.Z.); 19020852047@usx.edu.cn (L.Z.); lich@usx.edu.cn (C.L.); yuyanfei@usx.edu.cn (Y.Y.); 2 Shaoxing Key Laboratory of Interaction between Soft Soil Foundation and Building Structure, Shaoxing 312000, China

**Keywords:** geosynthetics, EPS lightweight soil, dynamic elastic modulus, damping ratio, series-parallel model

## Abstract

In recent years, expanded polystyrene (EPS) lightweight soil has been widely used as subgrade in soft soil areas because of its light weight and environmental protection. This study aimed to investigate the dynamic characteristics of sodium silicate modified lime and fly ash treated EPS lightweight soil (SLS) under cyclic loading. The effects of EPS particles on the dynamic elastic modulus (*E_d_*) and damping ratio (*λ*) of SLS were determined through dynamic triaxial tests at various confining pressures (σ_3_), amplitudes, and cycle times. Mathematical models of the *E_d_* of the SLS, cycle times, and σ_3_ were established. The results revealed that the EPS particle content played a decisive role in the *E_d_* and *λ* of the SLS. The *E_d_* of the SLS decreased with an increase in the EPS particle content (EC). The *E_d_* decreased by 60% in the 1–1.5% range of the EC. The existing forms of lime fly ash soil and EPS particles in the SLS changed from parallel to series. With an increase in σ_3_ and amplitude, the *E_d_* of the SLS gradually decreased, the *λ* generally decreased, and the *λ* variation range was within 0.5%. With an increase in the number of cycles, the *E_d_* of the SLS decreased. The *E_d_* value and the number of cycles satisfied the power function relationship. Additionally, it can be found from the test results that 0.5% to 1% was the best EPS content for SLS in this work. In addition, the dynamic elastic modulus prediction model established in this study can better describe the varying trend of the dynamic elastic modulus of SLS under different σ_3_ values and load cycles, thereby providing a theoretical reference for the application of SLS in practical road engineering.

## 1. Introduction

Soft ground is widespread in coastal areas and cities. It is known that the insufficient bearing capacity of a soft soil foundation is easily subjected to excessive settlement under the action of traffic load, and weight of subgrade and pavement structure. This eventually leads to engineering problems such as bridge head uplift and pavement cracking [1,2,3,4]. Reducing subgrade dead weight is one of the effective methods for reducing foundation settlement, so lightweight geomaterials have attracted wide interest.

Expanded polystyrene (EPS) granules, EPS blocks, and waste tire scraps are commonly used lightweight geomaterials [5,6,7]. Compared with EPS blocks, EPS particles are more compatible and can fill structures with arbitrary shapes [8]. Therefore, EPS particles are widely used in the field of civil engineering, especially as the aggregate of lightweight soil with advantages such as low density, thermal insulation, good compatibility, wide sources, and low price [9,10,11]. EPS granular lightweight soil is a new material formed by mixing soil, EPS granules, and a curing agent in a certain proportion with water. Using this material as subgrade filler can reduce the basic weight and seismic load of roads, thus improving the stability of the foundation [12,13,14]. However, EPS has low stiffness, low strength, and high compressibility, and its use as a lightweight aggregate may weaken the mechanical properties of soil materials, including strength characteristics and deformation characteristics [15]. In the past, many quantitative studies have been carried out on the physical and static characteristic of EPS lightweight soil, including compressibility, permeability, unconfined compressive strength (UCS), shear strength, California bearing ratio, stress–strain behavior, etc. [16,17,18,19,20,21,22]. The results show that the increase in EPS particle content (EC) reduces the density of EPS granular lightweight soil, and at the same time leads to the decline of various strength indexes and deformation properties of EPS granular lightweight soil [23,24]. This is because EPS particles themselves have high porosity, with air accounting for 98% of their volume [25]. Therefore, many studies use cement, lime, fly ash, and other curing agents to improve the strength of the soil skeleton in the subgrade filler, and use EPS granules to reduce the density of the subgrade filler, in order to obtain lightweight and high-strength subgrade filler materials [11,24,26,27,28,29]. Current studies have shown that a traditional inorganic chemical curing agent is also suitable for EPS light soil.

However, in actual engineering applications, the dynamic traffic stress impacts EPS lightweight soil as a subgrade material; hence, it is crucial to study its dynamic properties [30]. In recent years, a large number of studies on dynamic characteristics of cement-based EPS lightweight soil have appeared. El-Sherbiny et al. [31] studied the dynamic characteristics of EPS lightweight sand under large and small strains through cyclic triaxial and resonant column tests. It was found that as the EC increased, the shear stiffness of the sample decreased, whereas the material damping ratio remained mostly unaltered under mild shear strain. To determine the stress–strain characteristics of EPS lightweight mixed soil (RDSEM) under cyclic loading, Zhou et al. [32] evaluated the effects of cement, EPS particles, and confining pressure (σ_3_) through a dynamic triaxial test. Zhou et al. [33] studied the effects of different cement contents, EC, and σ_3_ on the dynamic elastic modulus (*E_d_*) and damping ratio (*λ*) of EPS granular lightweight clay (LCES) through a dynamic triaxial test, and compared them with the dynamic characteristics of clay. Gao et al. [34] studied the changes of stiffness and damping ratio of EPS lightweight soil with EPS, cement content, and confining pressure under low strain. 

The use of environmentally friendly curing agents is the future trend. Therefore, based on previous studies, lime, fly ash, and sodium silicate were used as the combined curing agent in this study [35,36]. In addition, although the influence of different mixing ratios on the dynamic characteristics of EPS lightweight soil (SLS) has been discussed in previous limited experiments, there is no study on the dynamic characteristics of EPS lightweight soil treated with sodium silicate–lime–fly ash (SLS). Therefore, this study analyzed the effects of EPS particle content, confining pressure, and strain amplitude on the dynamic elastic modulus and damping ratio of SLS. A series-parallel model is also proposed to analyze the variation of *E_d_* with EC. Finally, an empirical model is proposed to enable engineers to predict *E_d_* under different confining pressures and cycle times.

## 2. Materials and Methods

### 2.1. Materials

**Figure 1:**
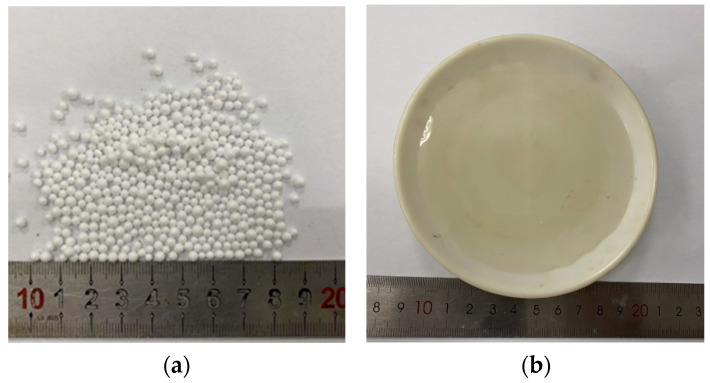
Test material: (**a**) Photograph of EPS particles; (**b**) Sodium silicate solution.

Lime, fly ash, sodium silicate, and soil were used in this test. The soil (Table 1) and fly ash (Table 2) used in the tests were obtained from Shaoxing, Zhejiang Province, China. Lime was quicklime powder produced in Xinyu, Jiangxi Province, China (Table 3). The EPS particles used in the test were obtained from a furniture factory in China. The particle sizes of EPS ranged from 2 to 3 mm, the bulk density was 0.018 g·cm^−3^, and the pure particle density was 0.0314 g·cm^−3^, as shown in Figure 1a. The sodium silicate used in the test was obtained from Bengbu Jingcheng Chemical Co., Ltd., Bengbu City, Anhui Province, China, with a concentration of 40% and modulus of 3.2 (Figure 1b).

### 2.2. Test Instrument and Test Scheme

**Figure 2:**
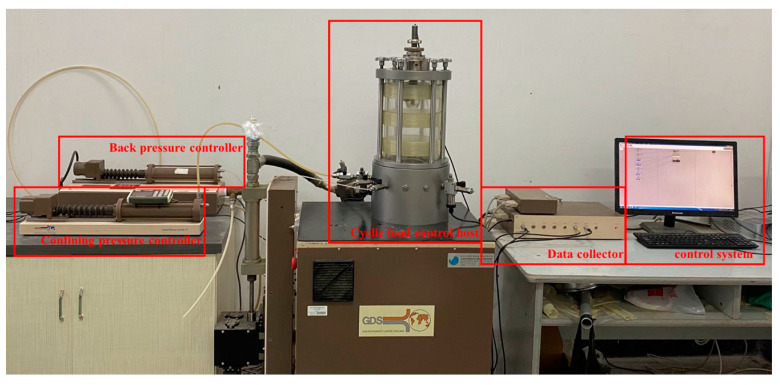
Dynamic triaxial test instrument.

The dynamic triaxial instrument used in this test is produced by GDS Company. The instrument consists of five parts including the confining pressure controller, back pressure controller, cyclic load control host, data collector, and control system, as shown in Figure 2.

**Figure 3:**
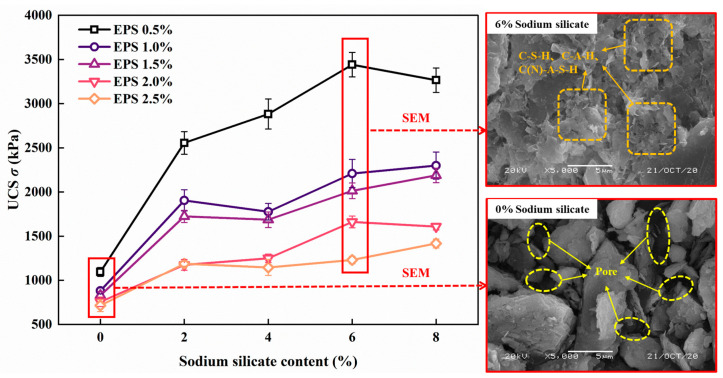
Unconfined compression test and microscopic test diagram of SLS.

According to the “Technical Guidelines for Construction of Highway Roadbases” JTG/T F20—2015 [37] and reference [38], the proportion of lime and fly ash was 1:3, the mass of lime and fly ash accounted for 20% of the mass of dry soil, the moisture content was 50%, and the masses of EPS particles were 0.5%, 1%, 1.5%, 2%, and 2.5% of the mass of dry soil. In addition, through an unconfined compression test and scanning electron microscope test, different proportions of sodium silicate were added to the EPS granular lightweight soil with lime and fly ash. The results are shown in Figure 3. It was found that the UCS reached a maximum when the sodium silicate content was 6–8%. The SEM test showed that, compared with no sodium silicate, after adding 6% sodium silicate, the internal hydration products (calcium silicate (C-S-H), calcium aluminate (C-A-H)) and gelling products (hydrated calcium aluminosilicate (sodium) (C (N)-A-S-H)) increased significantly, the porosity decreased significantly, and the strength improved [11,35]. Considering the comprehensive economy, the quality of sodium silicate was 6% of the mass of the dry soil. Based on this, dynamic triaxial tests with different confining pressures, amplitudes, and cycle times were conducted. The specific test schemes are listed in Table 4. In this test, displacement was used as the control mode to load it. The loading waveform was sinusoidal, and 50 points were collected for each cycle. This test simulated the traffic cyclic load, and the vibration frequency was 1 Hz [39]. As the axial displacement cyclic loading mode was adopted, the dynamic characteristics changed mainly at the initial stage of cyclic loading; therefore, the maximum number of cycles selected for this study was 100.

### 2.3. Specimen Preparation

**Figure 4:**
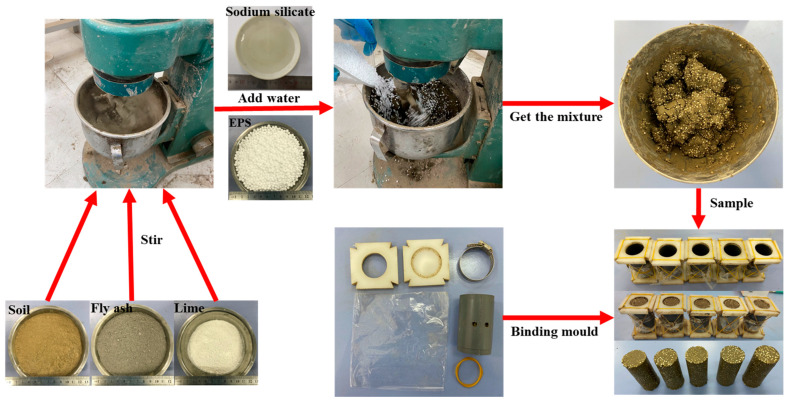
Sample preparation process.

As a large number of EPS particles were mixed in light soil, the sample preparation method of layered compaction will lead to deformation and rebound of EPS particles, which will eventually lead to soil rebound, cracking, and other problems. Therefore, the sample was prepared using manual layered vibration molding. The sample preparation process is as follows (see Figure 4):

(1) The soil was dried, crushed, and sieved in a 2 mm sieve before being mechanically combined with lime and fly ash until it was uniformly dispersed. Simultaneously, water and the sodium silicate solution were mixed and stirred evenly.
(2) The mixture of sodium silicate solution and water was poured into the mixture of lime, fly ash, and soil and stirred for 3 min. Finally, it was poured into weighed EPS particles and stirred until the EPS particles were evenly distributed in the soil.
(3) The mixture was loaded into a binding sample maker (D = 39.1 mm, H = 80 mm) 3 times and vibrated 50 times after each loading. The mixture was allowed to stand for 3 h after being vibrated 3 times. A scraper was then used to smooth the surface of the sample. The bottom plates at both ends and the sample maker were removed, and the sample was demolded, then obtained.
(4) The sample was placed in a standard curing room for 28 d. During curing, the temperature in the standard curing room fluctuated at 20 ± 2 °C, and the humidity was always above 90%.

The weight of the sample after curing is shown in Table 5.

## 3. Test Results and Discussion

**Figure 5:**
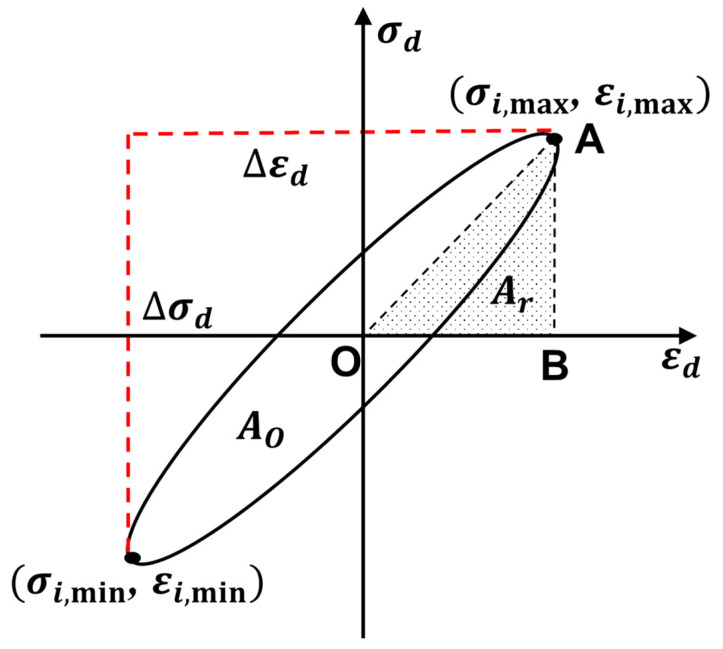
Schematic diagram of the typical hysteresis loop.

*E_d_* and *λ* mostly represent the dynamic properties of soil [40,41]. A series of hysteresis loops can be obtained under the action of axial cyclic loads [42]. Figure 5 shows a typical hysteresis loop [43,44], where *E_d_* is the ratio of the maximum stress difference (Equation (1)) to the maximum strain difference (Equation (2)) under the action of a cyclic load [45,46]. The calculation formula is shown in Equation (3).

(1) $$\Delta \sigma_{d}=\sigma_{i,max}-\sigma_{i,min}$$

(2) $$\Delta \varepsilon_{d}=\varepsilon_{i,max}-\varepsilon_{i,min}$$

(3) $$E_{d}=\Delta \sigma_{d}/\Delta \varepsilon_{d}$$

where $\sigma_{i,max}$ and $\sigma_{i,min}$ represent the maximum and minimum dynamic stresses corresponding to the current hysteresis loop, respectively (kPa). $\varepsilon_{i,max}$ and $\varepsilon_{i,min}$ represent the maximum and minimum dynamic strains corresponding to the current hysteresis loop, respectively (%). *E_d_* is the dynamic modulus of elasticity (MPa). Δσ represents the dynamic stress (kPa), and Δε is the dynamic strain (%).

The damping of soil reflects the characteristics of the dissipation of deformation energy with vibration due to retardation under dynamic loads. The commonly used damping ratio *λ* indicates its size [47,48]. The larger the *λ*, the larger the soil mass is, and the greater is its ability to resist vibration attenuation. Equation (4) is the formula for *λ*:

(4) $$\lambda =A_{0}/\left(4\pi A_{r}\right)$$

where *λ* is the damping ratio, *A*_0_ is the area enclosed by the dynamic stress and dynamic strain hysteretic loops, and *A_r_* is the area of the triangular AOB, as shown in Figure 5.

### 3.1. Influence of EPS Particle Content on E_d_ and λ

#### 3.1.1. Effect of EPS Particle Content on *E_d_*

**Figure 6:**
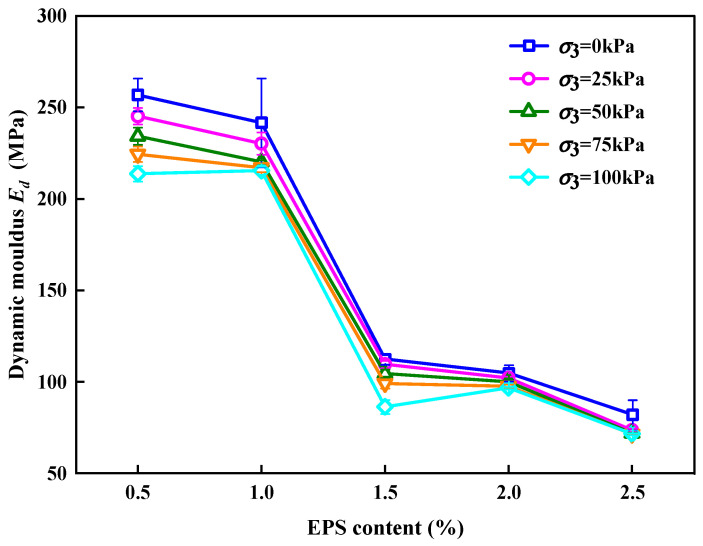
Relationship between EC and *E_d_*.

Figure 6 shows the effect of EC on *E_d_*. It can be observed that the *E_d_* of the SLS had the same change rule under different constraining pressures; that is, *E_d_* decreased in a “Z” shape with an increase in EC, indicating that EC plays a decisive role in the *E_d_* of the SLS. This was because the elastic modulus of EPS particles was smaller than that of sodium silicate solution modified lime fly ash soil (LFS). With an increase in EC, the overall elastic modulus decreased. After cyclic loading, EPS particles and LFS were unable to deform, so they separated along the interface and reduced *E_d_* [49] When EPS particles were mixed into the LFS, the LFS produced pores. The larger the number of EPS particles, the larger the number of pores [50]. The *E_d_* decline mechanism was analyzed in three stages.

(1) Slow-descent stage

When EC < 1%, there were relatively few EPS particles in the SLS and more consolidated soil in the SLS of the same volume. Lime fly ash and sodium silicate underwent hydration, ion exchange, crystallization, and other reactions during maintenance in the soil. The generated cement filled the pores and refined the pore size, thereby making the overall skeleton more compact [45,46,47,51,52,53]. Under the action of cyclic loading, the damage to the overall skeleton was not obvious; therefore, the *E_d_* of the SLS decreased slowly with an increase in EC.

(2) Rapid descent stage

When 1% < EC < 1.5%, the LFS in the SLS was relatively small. Under the action of a cyclic load, the overall skeleton was gradually damaged and loosened, and then gradually deformed. Therefore, an increase in EC led to a rapid decrease in the *E_d_* of the SLS, and the *E_d_* decreased by 60%.

(3) Slow decline to stabilize stage

When EC > 1.5%, more EPS particles were present in the SLS. Under cyclic loading, the entire skeleton was more likely to be damaged, resulting in less constraint of the skeleton on the EPS and easier EPS deformation. The elastic properties of EPS particles played a role; thus, the *E_d_* of the SLS gradually decreased and stabilized at the modulus of the EPS particles with an increase in EC.

#### 3.1.2. Series Parallel Model for *E_d_* of SLS 

**Figure 7:**
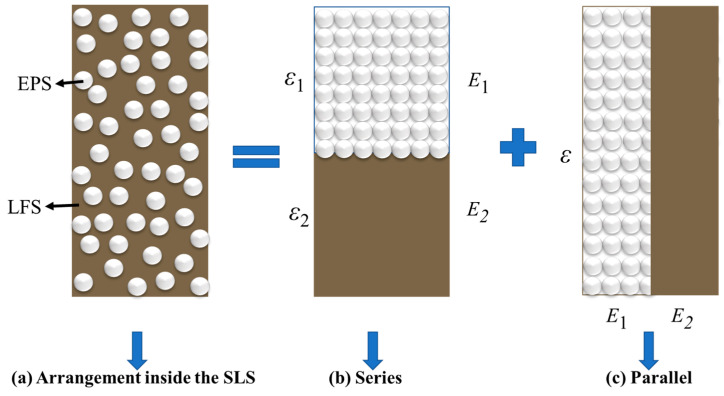
Arrangement of EPS particles and lime fly ash soil in the sample.

EPS particles and LFS are two kinds of materials with different physical properties. The elastic modulus E1 of EPS particles and E2 of LFS are not equal, and both resist deformation together. In addition, in general physical phenomena series and parallel are common forms of material arrangement. For example, the effective thermal conductivity system of porous material composed of any two phases is determined by the thermal conductivity of the two phases and their distribution. Therefore, when studying the *E_d_* of SLS, the arrangement form of EPS particles and LFS is regarded as a linear combination of series and parallel [54,55]. Assuming that the SLS sample was made up of a series of springs, the sample could contain series and parallel forms of LFS and EPS particles, as shown in Figure 7.

As shown in Figure 7b, the EPS particles in the SLS were connected in series with the LFS, and their elastic modulus is expressed in Equation (5).

(5) $$E_{s}=\frac{\sigma }{\varepsilon_{1}+\varepsilon_{2}}=\frac{\sigma }{\frac{\sigma }{E_{1}}+\frac{\sigma }{E_{2}}}=\frac{1}{\frac{1}{E_{1}}+\frac{1}{E_{2}}}$$

where *E_s_* is the elastic modulus in the series, $\varepsilon_{1}$ is the strain of the EPS particles, and $\varepsilon_{2}$ is the strain of the LFS. *E*_1_ is the elastic modulus of the EPS particles (generally, 2.5 MPa) [50,56], and *E*_2_ is the elastic modulus of the LFS. The elastic modulus was 311.5 MPa based on the preliminary test. Based on Equation (5), *E_s_* = 2.48 MPa.

As shown in Figure 7c, the EPS particles in the SLS were connected in parallel with the LFS, and their elastic modulus is expressed in Equation (6).

(6) $$E_{p}=\frac{\sigma }{\varepsilon }=\frac{E_{1}\cdot \varepsilon +E_{2}\cdot \varepsilon }{\varepsilon }=E_{1}+E_{2}$$

where *E_p_* is the elastic modulus in parallel mode and ε is the strain of the SLS sample. Based on Equation (6), *E_p_* = 314 MPa.

In the actual test, both series and parallel modes co-existed in the SLS sample, as shown in Figure 7a. Assuming that they existed in the form of a linear superposition, the elastic modulus of the SLS is expressed in Equation (7).

(7) $$E=\eta \cdot E_{s}+\left(1-\eta \right)\cdot E_{p}$$

where *η* denotes the proportion of the series mode. When *η* = 1, the particles of the LFS and EPS in the SLS existed in series, and their elastic moduli were small. When *η* = 0, the LFS and EPS particles in SLS existed in parallel, and their elastic moduli were large. The value of *η* is related to the EC and load type and was calculated using the experimental data.

**Figure 8:**
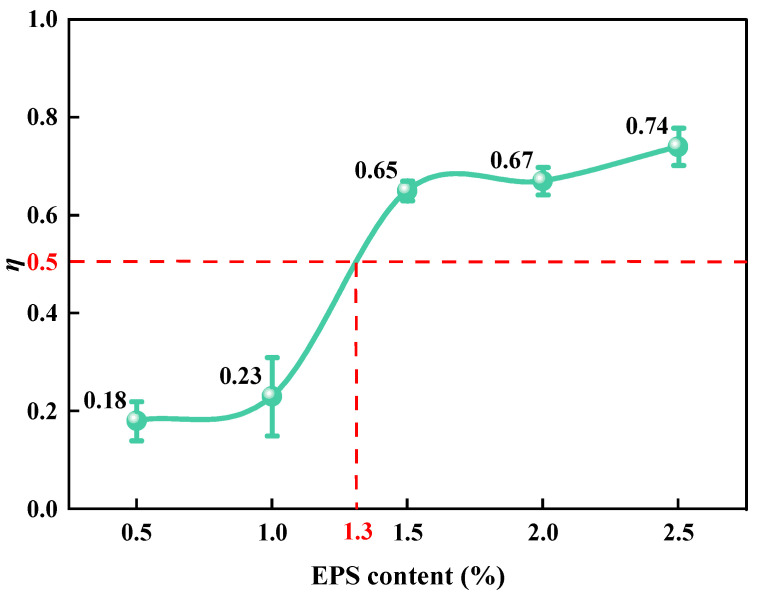
Relationship between EC and *η*.

The value of *η* was obtained using Equation (7). Under different EC conditions, the proportions of SLS samples in series and parallel were obtained. The relationship between *η* and EC is illustrated in Figure 8. It can be observed that when EC was less than 1%, the value of *η* was less than 0.23, and the change was slight. The EPS and LFS in the sample mostly existed in parallel. When the EC increased from 1% to 1.5%, the *η* value increased rapidly from 0.23 to 0.65, and the LFS and EPS particles in the sample changed mainly from being in parallel to being in series. When the EC value was greater than 1.5%, the *η* value increased gradually to 0.74 with an increase in EPS content. The LFS and EPS particles mainly existed in series mode in the sample. The series and parallel models reasonably explained how the *E_d_* in Figure 6 decreased in three stages of the “Z” shape with an increase in EC.

#### 3.1.3. Effect of EPS Particle Content on λ

**Figure 9:**
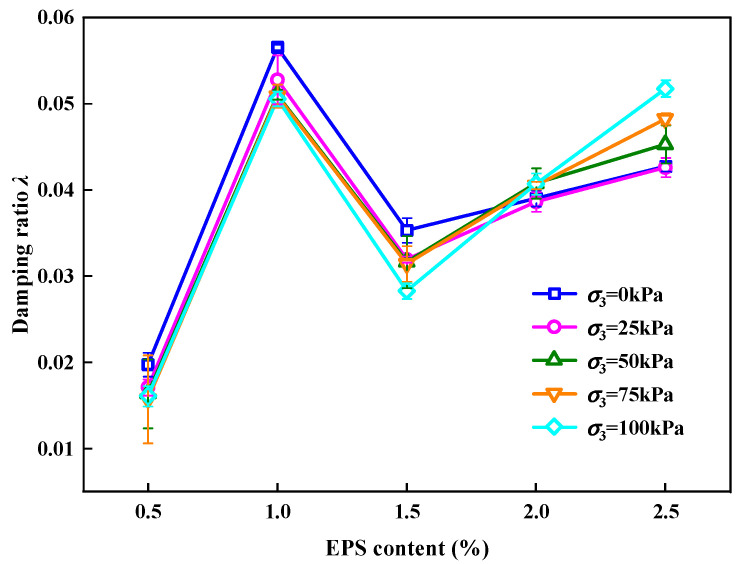
Relationship between EC and *λ*.

Figure 9 shows the impact of EPS particles on the *λ* value of the SLS. It can be observed that for SLS under different σ_3_ environments, the changing trend of *λ* is an “N” shape with the content of EPS particles. This shows that EC plays an important role in the *λ* of the SLS. The change mechanisms were analyzed in three stages.

(1) When EC < 1%, *λ* first increased with an increase in EC. This was because, as the cyclic load progressed, the overall skeleton loosened, and *λ* increased because the energy dissipated by friction increased.
(2) When 1% < EC < 1.5%, *λ* decreased with an increase in EC. This was because, as the cyclic load progressed and when the EC was approximately 1.5%, the EPS particles in SLS were completely compacted. The slip and dislocation of particles in the SLS were reduced, and *λ* decreased owing to the reduction in energy dissipated by friction and elastic deformation of EPS particles.
(3) When EC > 1.5%, *λ* increased with increasing EPS particle content. This was caused by the increase in the EPS particle content, which replaced the LFS. As the pore structure in the SLS increased, the LFS volume decreased, and more energy was lost in the propagation of stress waves in the SLS, which contained a large proportion of EPS particles. As the cyclic loading continued, there were still many EPS particles that were not compacted. This portion of the EPS particles produced elastic deformation and consumed energy; thus, *λ* increased again.

### 3.2. Influence of Confining Pressure on E_d_ and λ

#### 3.2.1. Effect of Confining Pressure on *E_d_*

**Figure 10:**
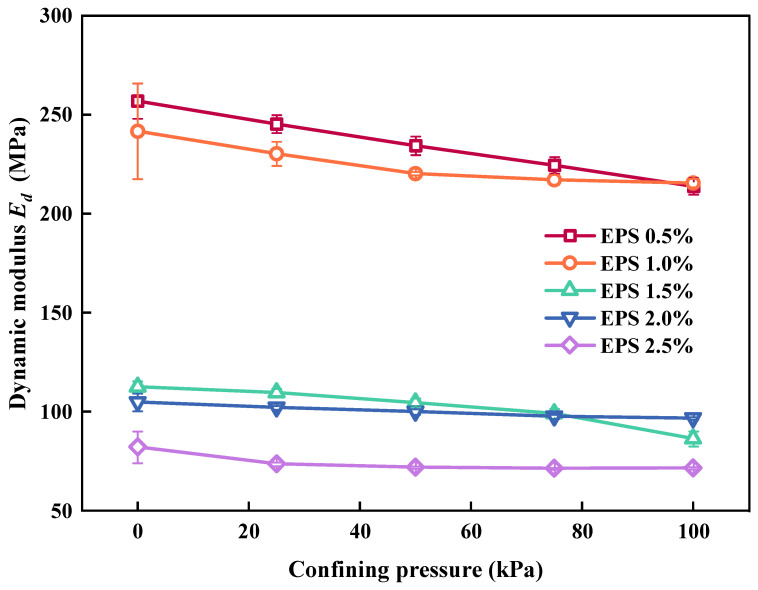
Relationship between confining pressure and *E_d_*.

Figure 10 shows the effect of σ_3_ on *E_d_*. When EC was the same, *E_d_* follows the σ_3_ raise and lower. This was caused by the fact that increasing the confining pressure decreases the elastic characteristics of EPS particles, and that cyclic loading causes an increase in irreversible plastic deformation. The deformation of the SLS increased, resulting in a decrease in *E_d_*. However, when EC was greater than 2%, the decrease in *E_d_* with an increase in the confining pressure was not obvious. This was because the EC was too high and the increase in the confining pressure could not effectively inhibit the elastic properties of the EPS particles. There were still some EPS particles in the SLS that could provide elastic properties; therefore, the reduction in *E_d_* was not apparent.

#### 3.2.2. Effect of Confining Pressure on *λ*

**Figure 11:**
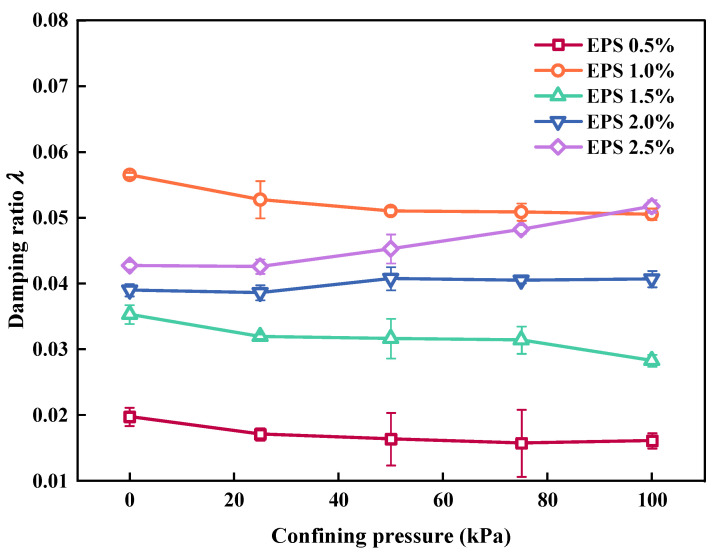
Relationship between confining pressure σ_3_ and *λ*.

Figure 11 shows the effect of σ_3_ on *λ*. When EC remained constant, the *λ* value of the SLS decreased as σ_3_ increased; however, the total variation range of *λ* with σ_3_ was minimal, falling within 0.5%. This was consistent with the test results of Lu [3]. This was because the interior of the SLS was more tightly packed due to the increase in σ_3_. Thus, there was less sliding and dislocation between them and less friction between the various particles. The value of *λ* decreased because there was less energy lost by the stress wave during its propagation.

### 3.3. Influence of Amplitude on E_d_ and λ

#### 3.3.1. Effect of Amplitude on *E_d_*

**Figure 12:**
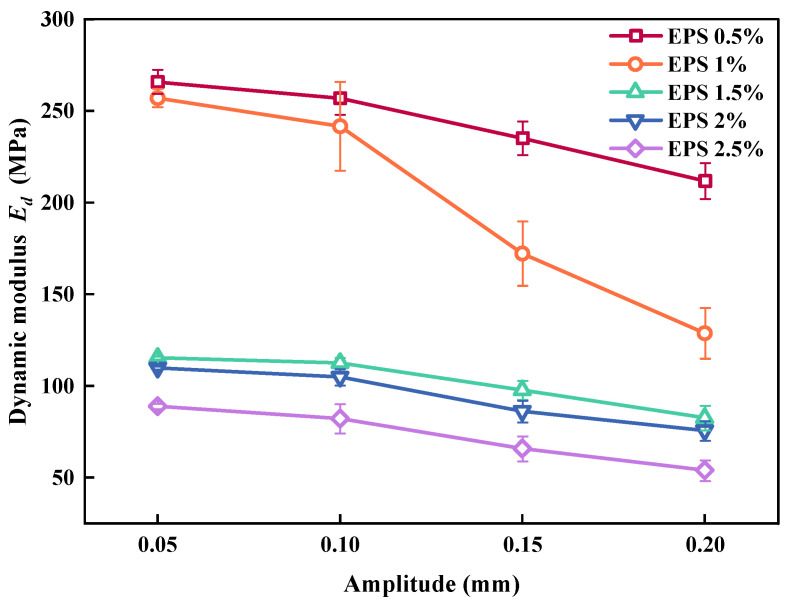
Relationship between amplitude and *E_d_*.

Figure 12 shows the effect of the amplitude on *E_d_*. It can be observed that when EC was the same, *E_d_* decreased with an increase in amplitude. This was because the increase in amplitude (although small amplitude was not sufficient to destroy the SLS) loosened the overall skeleton of the SLS and made the EPS particles easier to compact. The greater the amplitude, the greater the deformation of SLS, resulting in a decrease in *E_d_*. The *E_d_* of SLS with high EC decreased relatively slowly, with an increase in amplitude owed to excessive EC. Under cyclic loading, the increase in amplitude did not effectively inhibit the elastic potential energy of the EPS particles. The deformation of the SLS decreased slowly, indicating that *E_d_* decreased relatively slowly with a decrease in amplitude.

#### 3.3.2. Effect of Amplitude on *λ*

**Figure 13:**
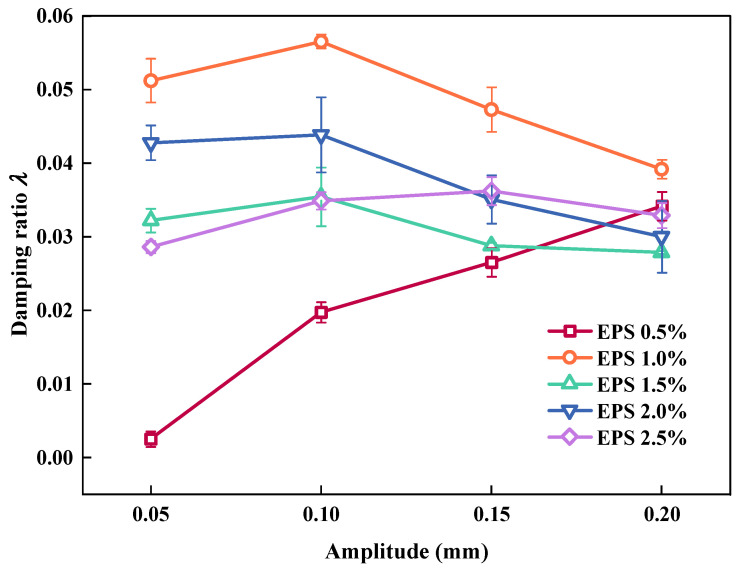
Relationship between amplitude and *λ*.

Figure 13 shows the effect of amplitude on *λ*. As can be observed, the *λ* of the SLS decreased as the amplitude increased, and the variation range was very limited. This was caused by the increase in the amplitude. During cyclic loading, the interior of the SLS was gradually compacted, and the slip and dislocation of particles in the SLS were reduced, reducing the energy consumed by friction. When EC was 0.5%, the *λ* value of the SLS increased rapidly with an increase in amplitude. This was because when EC was small there was more LFS in the same volume of the SLS. With an increase in amplitude, under the action of a cyclic load, the overall skeleton loosens. Because of the increase in energy dissipated by friction, more energy was lost during the transmission process of the stress wave, resulting in an increase in *λ*.

### 3.4. Influence of Cycle Times on E_d_ and λ

**Figure 14:**
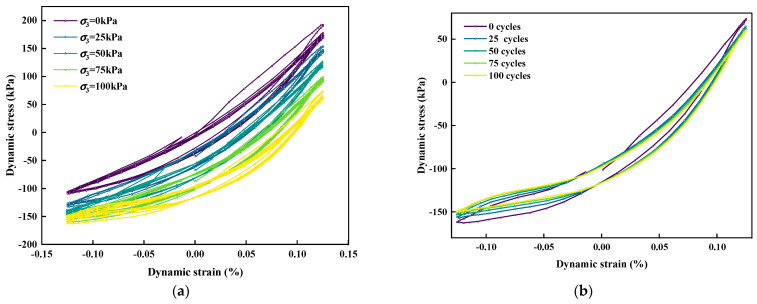
Hysteretic curve under different confining pressure and different numbers of cycles: (**a**) Stress−strain curves under cyclic loading with different confining pressures; (**b**) variation diagram of the hysteretic curve with cycle count under a confining pressure of 100 kPa.

**Figure 15:**
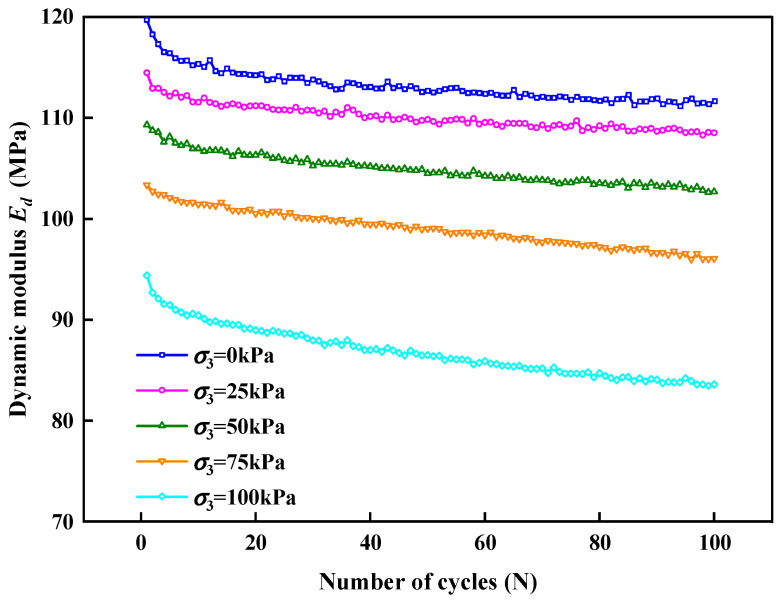
Variation of *E_d_* with the number of cycles applied under various σ_3_ values.

Figure 14 shows hysteretic curves under different confining pressures and different cycles. Figure 14a shows the stress–strain curves (hysteretic loops) of the cyclic load when the EC was 1.5% under different confining pressures. The dip angle of the hysteresis ring can represent the relaxation degree of soil under the action of axial dynamic stress, and can reflect the change of soil stiffness under the action of dynamic load and the cumulative damage degree in the sample [57]. That is, the change in the inclination of the hysteresis loop can reflect the *E_d_* change of the SLS under cyclic loading. The smaller the inclination, the smaller the *E_d_*. As shown in Figure 14a, with an increase in σ_3_, the hysteresis loop gradually moves downward and the inclination decreases; therefore, the *E_d_* of the SLS decreased with an increase in σ_3_. This conclusion is consistent with that in the previous text. Figure 15b shows the hysteretic loops of the SLS with the number of cycles applied by the cyclic load when σ_3_ = 100 kPa. It is clear that as the cyclic load was applied with more cycles, the inclination of the hysteretic loop gradually decreased and its *E_d_* diminished.

Taking an EPS content of 1.5% as an example, the *E_d_* of each hysteretic loop in a process of 100 cycles was calculated, and the change in the *E_d_* of the SLS with the number of cycles under different σ_3_ was obtained, as shown in Figure 15. At the initial stage of the cyclic loading, the *E*_d_ of the SLS under different σ_3_ values decreased rapidly with an increase in the number of cycles and then tended to stabilize gradually. This is because at the initial stage of the cyclic loading, after the dynamic load was applied the unrecoverable plastic deformation of the SLS increased, the deformation of EPS particles continued to increase, and Ed decreased rapidly. With the increase in the number of cycles and the limitation of the amplitude (0.1 mm), the deformation of the EPS particles did not change and tended to stabilize gradually. The same phenomenon has also appeared in other studies. The researchers believe that this phenomenon is due to the rapid deformation of EPS during the initial stage of strain cycling, resulting in a rapid change in elastic modulus. As the strain cycle continues, the deformation phase difference of EPS particles causes the deformation of EPS particles to not occur simultaneously with the cyclic strain, so it tends to stabilize [43]. 

Based on the previous analysis, the *E_d_* of the SLS first decreased rapidly, then tended to stabilize with an increase in the number of loading cycles, and decreased with an increase in σ_3_. Based on the above rules [58], the *E_d_* and cycle times under various σ_3_ values satisfied the power function relationship, as shown in Equation (8).

(8) $$E_{d}=a\cdot N^{b}$$

where *a* and *b* are related to the confining pressure. Based on the relationship between the confining pressure and *E_d_* shown in Figure 15, the functional relationships between the parameters *a* and *b* and the confining pressure were determined, as shown in Equations (9) and (10), respectively.

(9) $$a=-0.2189p+120.32,\;R^{2}=0.96$$

(10) $$b=-0.0003p^{2}+0.0167p-1.6282,R^{2}=0.97$$

Equations (9) and (10) were substituted into Equation (8) to obtain Equation (11).

(11) $$E_{d}=\left(-0.2189p+120.32\right)N^{-0.0003p^{2}+0.0167p-1.6282}$$

where *N* is the number of cycles and *p* is the confining pressure.

**Figure 16:**
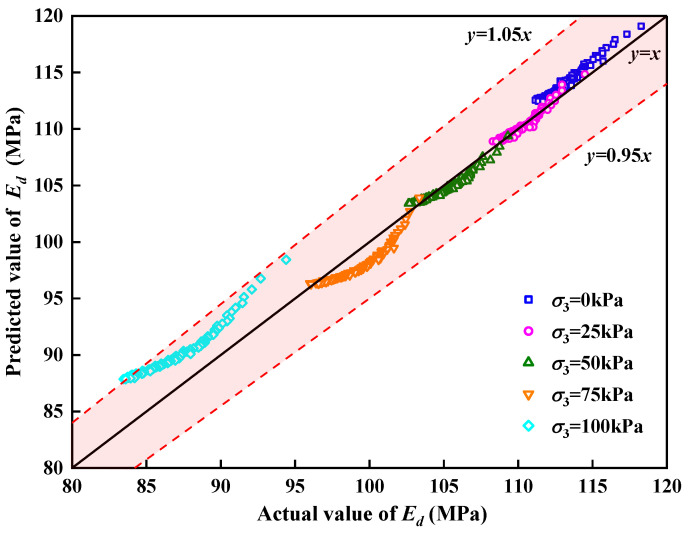
Comparison of actual and predicted *E_d_*.

The predicted value of the dynamic elastic modulus calculated using Equation (11) was compared with the actual value of the dynamic elastic modulus obtained from the tests. The comparison results are shown in Figure 16. It can be observed that the predicted value of *E_d_* was consistent with the experimental value, with an error within 5%. The model predicted the varying trend of the *E_d_* of the SLS with different confining pressures and cycle times.

**Figure 17:**
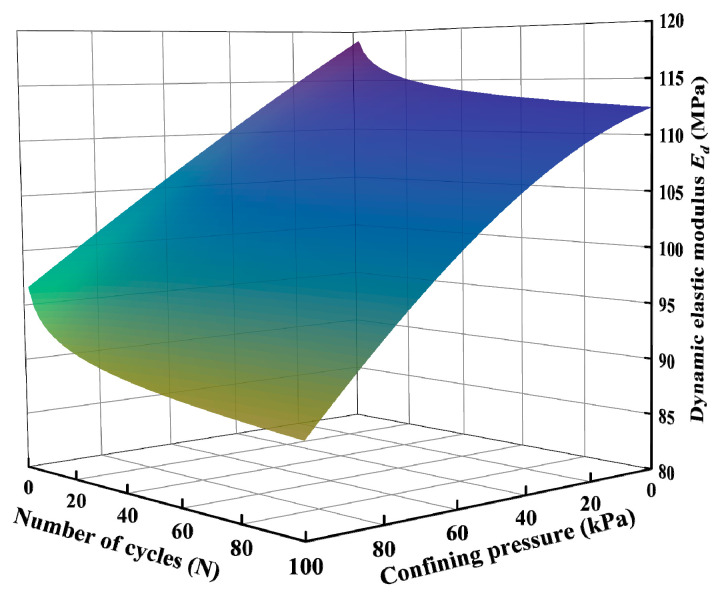
Model diagram of dynamic elastic modulus with confining pressure and cycle times.

The varying pattern of the *E_d_* was obtained using Equation (11), as shown in Figure 17. It was found that with an increase in confining pressure and cycle times, the *E_d_* of the SLS gradually decreased, and the effect of the confining pressure was relatively obvious. In the yellow area in Figure 17, the *E_d_* of the SLS tended to be minimal. When the number of cycles continued to increase, the *E_d_* tended to stabilize.

## 4. Conclusions

Adding EPS particles into subgrade filling material can effectively reduce the density of filling material, improve the stability of the original subgrade, and reduce the uneven settlement of soft subgrade. In this study, the effects of EPS particle content, confining pressure, amplitude, and cycle times on the *E_d_* and *λ* of SLS were evaluated by using a dynamic triaxial test, which provides theoretical basis and data support for the application of SLS in road engineering. The main conclusions are as follows:

(1) The EPS particle content played a decisive role in the *E_d_* and *λ* of the SLS, and the optimal dosage of EPS particles is between 0.5% and 1%. When σ_3_ and amplitude are constant, *η* increases with the increase of EC, and the layout of EPS and LFS in SLS is transferred from parallel to series. When EC increased from 1% to1.5%, *E_d_* decreases by 60%, resulting in a Z-shaped decline in *E_d_* of SLS. The *λ* of SLS is N-shaped with the increase in EPS particle content.
(2) When EC was constant, the *E_d_* of the SLS decreased with an increase in σ_3_ and amplitude; however, the effect of change in confining pressure and amplitude on *E_d_* was not obvious under high EC values. This was because the number of EPS particles was too high and some EPS particles in the SLS provided elastic properties; thus, the reduction in *E_d_* was not obvious. The *λ* of the SLS generally decreased with an increase in the confining pressure and amplitude, and the variation range was small (within 0.5%).
(3) The *E_d_* of the SLS satisfied the power function relationship with the number of load actions. The *E_d_* value predicted by the model established in this study was consistent with the actual value, and the error was within 5%. The model predicted well the varying trend of the *E_d_* under various σ_3_ values and cycle times.

## Figures and Tables

**Table 1:** Main physical and mechanical indices of subgrade soil.

Plastic Limit (%)	Liquid Limit (%)	Natural Density (g·cm^−3^)	Moisture Content (%)	Organic Content (%)
24.60	40.90	1.84	50	6.50

**Table 2:** Main chemical composition of fly ash.

Al_2_O_3_ (%)	SiO_2_ (%)	SO_3_ (%)	CaO (%)	TiO_2_ (%)	Fe_2_O_3_ (%)	Other (%)
23.45	22.94	6.35	33.7	3.56	7.26	2.74

**Table 3:** Main chemical composition of lime.

CaO (%)	MgO (%)	Fe_2_O_3_ (%)	Al_2_O_3_ (%)	TiO_2_ (%)	SiO_2_ (%)	Other (%)
89.42	1.76	0.18	0.25	7.47	0.58	0.34

**Table 4:** Test scheme.

Influence Factor	EPS Content (%)	Confining Pressure (kPa)	Amplitude (mm)
EPS particle content	0.5	0, 25, 50, 75, 100	0.1
	1		
	1.5		
	2		
	2.5		
Confining pressure	0.5	0, 25, 50, 75, 100	0.1
	1		
	1.5		
	2		
	2.5		
Amplitude	0.5	0	0.05, 0.1, 0.15, 0.2
	1		
	1.5		
	2		
	2.5		
Number of cycles	1.5	0, 25, 50, 75, 100	0.1

**Table 5:** Weight of the sample.

**EPS Content (%)**	**0.5**	**1**	**1.5**	**2**	**2.5**
**Weight of the sample (g)**	131.1	114.3	104.7	92.2	83.6

## Data Availability

Not applicable.
